# Adjacent segment degeneration

**Published:** 2012-11

**Authors:** Alireza Birjandi

**Affiliations:** ^a^Mashhad University of Medical Science, Mashhad, Iran.

## Abstract

Adjacent segment disease (ASD) is defined as degeneration that develops at mobile segments above or below a fused spinal segment and usually develops after spinal fusion or other back surgeries. Nearly 5 decades ago, the medical findings related to ASD were usually released in case reports as a relatively unusual complication of lumbar or lumbosacral fusions. Since the initial reports, ASD has been found to occur more often than the earlier predictions for its prospect incidence. It is now considered a potential long-term complication of spinal arthrodesis.

**Case:**

Numerous biomechanical studies have reported that the fusion process imposes significant amounts of stress on the adjacent segments. Although there has been a great deal of research performed on ASD, the prevalence and the cause of the disorder are not yet well documented. However, occurrence and medical findings of ASD are one of the main reasons for developing the motion-preserving techniques as an alternative to fusion surgery. Previous studies have proven the higher efficiency of the anterior lumbar interbody fusion combined with posterolateral lumbar fusion (ALIF+PLIF), as compared to the conventional method of posterolateral fusion alone in terms of outcome and cost-effectiveness.

**Keywords:**

Adjacent segment disease, Anterior lumbar interbody fusion, Posterolateral lumbar fusion, Spinal fusion


					Volume 4, Suppl. 1 Nov 2012
Publisher: Kermanshah University of Medical Sciences
URL: http://www.jivresearch.org
Copyright:
Congress of Iranian Neurosurgeons, 14-16 November 2012, Kermanshah, Iran
Paper No. 8
Adjacent segment degeneration
Alireza Birjandi a,*
a
Mashhad University of Medical Science, Mashhad, Iran.
Abstract:
Adjacent segment disease (ASD) is defined as degeneration that develops at mobile segments above or below a
fused spinal segment and usually develops after spinal fusion or other back surgeries. Nearly 5 decades ago, the
medical findings related to ASD were usually released in case reports as a relatively unusual complication of lumbar
or lumbosacral fusions. Since the initial reports, ASD has been found to occur more often than the earlier predictions
for its prospect incidence. It is now considered a potential long-term complication of spinal arthrodesis.
Case: Numerous biomechanical studies have reported that the fusion process imposes significant amounts of stress on
the adjacent segments. Although there has been a great deal of research performed on ASD, the prevalence and the
cause of the disorder are not yet well documented. However, occurrence and medical findings of ASD are one of the
main reasons for developing the motion-preserving techniques as an alternative to fusion surgery. Previous studies
have proven the higher efficiency of the anterior lumbar interbody fusion combined with posterolateral lumbar fusion
(ALIF+PLIF), as compared to the conventional method of posterolateral fusion alone in terms of outcome and cost-
effectiveness.
Keywords:
Adjacent segment disease, Anterior lumbar interbody fusion, Posterolateral lumbar fusion,
Spinal fusion
* Corresponding Author at:
Alireza Birjandi: Professor of neurosurgery, Mashhad University of Medical Science, Mashhad, Iran. E-mail: birjandibirjandi@gmail.com, (Birjandi A.).

				

